# *CytoJournal *joins 'open access' philosophy

**DOI:** 10.1186/1742-6413-1-1

**Published:** 2004-07-29

**Authors:** Vinod B Shidham, Anthony Cafaro, Barbara F Atkinson

**Affiliations:** 1 Co-Editor-in-chief, CytoJournal, Associate Professor, Medical College of Wisconsin, Milwaukee, WI, USA; 2 Assistant editor, CytoJournal, Assistant Professor, Medical College of Wisconsin, Milwaukee, WI, USA; 3 Co-Editor-in-chief, CytoJournal, Executive Dean, Kansas University Medical Center, Kansas City, KS, USA

**Keywords:** Cytopathology, cytojournal, Open Access, BioMed Central, cytopathology foundation

## Abstract

Welcome to *CytoJournal*! We would like to introduce you to your journal, one that is run by and for the scientific cytopathology community with incontestable benefits of Open Access, and support from Cytopathology Foundation, Inc. *CytoJournal *is a peer-reviewed, PubMed indexed, online journal, publishing research in the field of cytopathology and related areas, with world wide free access. Authors submitting to CytoJournal retain the copyright to their hard earned work.

## 

Welcome to CytoJournal ! We would like to introduce you to your journal, one that is run by and for the scientific cytopathology community with incontestable benefits of Open Access . *CytoJournal *is a peer-reviewed cytopathology journal is owned and supported by Cytopathology Fondation, Inc.  It is a PubMed indexed, online journal, publishing research in the field of cytopathology and related areas with world wide free access. Authors submitting to CytoJournal retain the copyright to their hard earned work.

## Why CytoJournal is needed

One could argue whether we need more journals in cytopathology, but without a shadow of doubt there is a global need for greater access to scientific information in this field. As an Open Access journal CytoJournal will meet this need, by removing subscription barriers.

Communication in general has been revolutionized in the last decade. With the emergence of the internet, entire libraries of scientific information are potentially just a mouse click away. Open Access to quality controlled, scientific information to the general public and scientific community alike is extremely valuable for harvesting the fruits of hard work by academicians.

However, to date little has been done to realize the potential of this technological revolution. It is now affordable to make our hard earned scientific information available to a much wider audience. Millions of students, teachers, physicians, scientists, general public, and other potential readers can have free access to the gold mine of this scientific information.

The traditional model for journal publication has performed an admirable job of disseminating research and advancing science. Unfortunately, it neither can fully utilize recent technology nor extend its benefits to the scientific community and the general public. Principally, why should scientific information generated by academicians and published without charge, not be freely available? Giving away the copyrights to the original research reports and then paying for access of the same material is paradoxically anachronistic.

With the advent of the internet and image digitization, the time has come for the development and implementation of a new model – a model beneficial to the scientific community and general public, alike. The recent adoption of an Open Access model by the Public Library of Science (PLoS) [1] and its recognition by other scientific organizations, including the National Institute of Health (NIH), has generated significant community interest. This has provided impetus for the creation of new journals such as *CytoJournal *with increasing support to open access philosophy by some of the existing leading journals.

The benefits of an online journal can include rapid turnaround time, real time publication, significant cost savings, and a reduction in the environmental burden engendered in the production and disposal of a print publication. Presumably in the future, the majority of research publications will be of this type [2].

Free flow of scientific information is crucial for advancements in diagnosis and management of diseases in both the developed and developing world. Simply providing a conduit of information is not enough. There is one matter involving the human element that must be dealt with – peer review. We do not see a future where authors post their manuscripts on the web without peer-review. Science requires that publications be properly vetted (peer-reviewed) and experimental findings logically presented.

In brief, standards must be maintained! This critical role is played by peer review. Acting on behalf of the scientific community, it is peer review that helps the scientific community distinguish between legitimate scientific work and quackery [3-7]. Thus, to maintain the quality and confidence, peer review will continue to be the keystone of scientific publishing.

**Figure 1:**
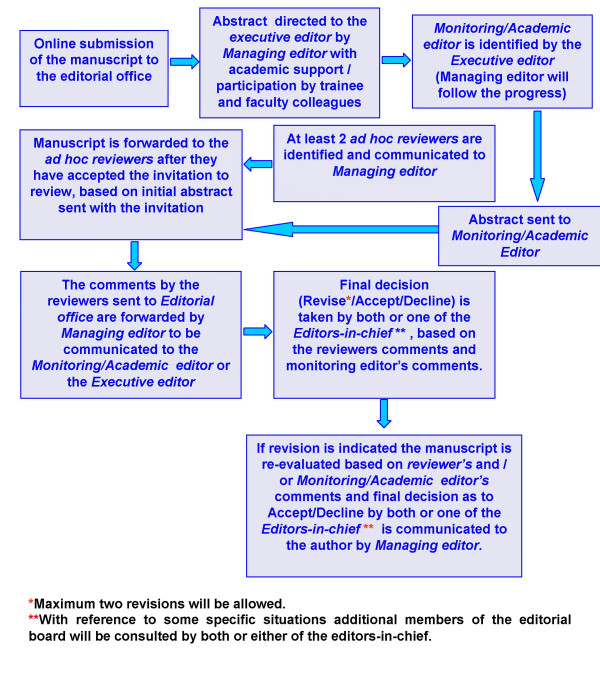
CytoJournal's online peer review process.

The peer-review process at CytoJournal combines the expertise of both professional editors, who are available to survey the broad landscape of cytopathology, and academic editors, who understand deeply the strengths and limitations of their specific area of research. Every article that is published in CytoJournal will be reviewed carefully by selected reviewers, in addition to evaluation by a managing/academic editor and professional editor, who work together throughout the editorial process. A summary of your peer review process is illustrated in Figure 1.

## Definition of Open Access

*CytoJournal's Open Access *policy changes the way in which articles are published. First, all articles become freely and universally accessible online, and so an author's work can be read by anyone, free of charge. Second, the authors hold copyright for their work and grant anyone the right to reproduce and disseminate the article, provided that it is correctly cited and no errors are introduced [8]. Third, a copy of the full text of each Open Access article is permanently archived in an online repository separate from the journal. *CytoJournal's *articles are archived in PubMed Central [9], the US National Library of Medicine's full-text repository of life science literature, and also in repositories at the University of Potsdam [10] in Germany, at INIST [11] in France and in e-Depot, the National Library of the Netherlands' digital archive of all electronic publications [12].

## Benefits of Open Access

Open Access has four broad benefits for science and the general public. First, authors are assured that their work is disseminated to the widest possible audience, given that there are no barriers to their work. This is accentuated by the authors being free to reproduce and distribute their work, for example by placing it on their institution's website. It has been suggested that free online articles are more highly cited because of their easier availability [13]. Second, the information available to researchers will not be limited by their library's budget, and the widespread availability of articles will enhance literature searching [14]. Third, the results of publicly funded research will be accessible to all taxpayers and not just those with access to a library with a subscription. As such, Open Access could help to increase public interest in, and support of, research. Note that this public accessibility may become a legal requirement in the USA if the proposed Public Access to Science Act is made law [15]. Fourth, a country's economy will not influence its scientists' ability to access articles because resource-poor countries (and institutions) will be able to read the same material as wealthier ones (although creating access to the internet is another matter) [16].

## Open Access Publishing Model and Finance

Open Access facilitates the transformation of scientific literature from rows of printed journals on library shelves to an instantly searchable archive of data. Liberating scientific literature from the vestiges of paper publication introduces the potential of various opportunities such as navigating, integrating, mining, annotating, and mapping connections in the high-dimensional space of scientific knowledge.

**Figure 2:**
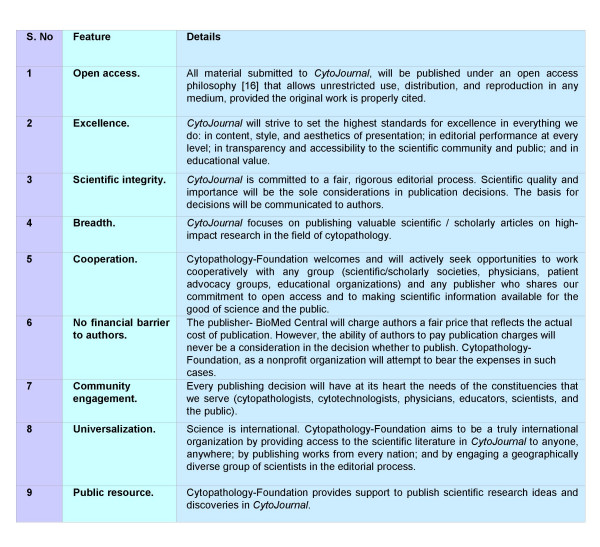
Core Principles of 'CytoJournal' and 'Cytopathology-Foundation'

To provide Open Access, *CytoJournal *will use a new business model. Our editorial expenses (managing peer review, providing editorial insight, and ensuring the highest production standards) will be supported by corporate sponsors through Cytopathology Foundation, Inc.  and honorary *pro bono *services by the academicians (Figure 2). The publication cost for our publishers, BioMed Central, will be recovered by imposing a modest charge (currently US$ 525) to authors for each article accepted for publication after peer review. There is no charge for the submission of a manuscript and therefore, if the manuscript is submitted but not accepted for publication, the authors will not be charged.

Our sponsors do not influence in any way the content of *CytoJournal *and it's editorial and publication decisions. However, they will be acknowledged and their links will be displayed on the home page of CytoJournal as a *thank you *note.

## Fee Waivers

We understand that there are many scientists who might wish to publish in our journal but do not have access to grant funds or institutional support. For such authors, a decision can be made by the Editors-in-Chief to waive publication fees. Furthermore, the fee will automatically be waived for authors from institutes that are members of BioMed Central . We never want our publication charges to be a barrier to publication and are committed to publishing any manuscript that our editors and reviewers deem to be appropriate for the journal; we will treat the costs of handling these papers as a fundamental expense of running a high-quality journal through support from Cytopathology Foundation Inc.

## Joining Forces

*CytoJournal *is your journal and you can lead the way. However, we have to face and overcome a few traditional obstacles. First, an unfamiliarity to the Open Access model. Second, lack of the benefits of an established "brand name" aura. Because of this, despite stringent standards and an extraordinary editorial team, *CytoJournal *may have an uphill task.

Private foundations with a commitment to science and education have contributed generously to the cause. Like any new business, Cytopathology Foundation Inc. needed to raise funds to cover our startup costs. Initial support from Cytopathology Foundation Inc. and the publisher, BioMed Central, has enabled us to launch *CytoJournal*. Other individuals and organizations  have also provided generous and welcome support. The start-up support made it possible to assemble an outstanding editorial board and staff, who have accomplished an extraordinary feat of launching this new premiere journal in cytopathology.

We wish to thank and applaud the efforts and spirit of the pioneering authors who chose to send their articles to *CytoJournal*. In the end, it is the contributions by authors like you that will make *CytoJournal *a collective success. We encourage you to participate in the future of *CytoJournal *by reading, citing, submitting manuscript, sending any suggestions, and joining the panel of core reviewers.

Bookmark the home page of *Cytojournal * for your quick reference! You may also link *CytoJournal *web site or recommend its linking through various other web sites.

## References

[B1] Public Library of Science (PLoS) Promotes the free exchange of scientific information. http://www.publiclibraryofscience.org.

[B2] Delamothe T, Smith R Editorial, Open access publishing takes off. BMJ.

[B3] Enserink M (2001). Peer review and quality: a dubious connection?. Science.

[B4] International Congress on Peer Review in Biomedical Publication. http://www.ama-assn.org/public/peer/peerhome.htm.

[B5] Fourth International Congress on Peer Review in Biomedical Publication. http://jama.ama-assn.org/issues/v287n21/toc.html.

[B6] Scholarly Publishing, Peer Review and the Internet. http://www.firstmonday.dk/issues/issue4_4/proberts/.

[B7] Implementing peer review on the net: Scientific quality control in scholarly electronic journals. http://www.ecs.soton.ac.uk/~harnad/Papers/Harnad/harnad96.peer.review.html.

[B8] BioMed Central Open Access Charter. http://www.biomedcentral.com/info/about/charter.

[B9] PubMed Central. http://www.pubmedcentral.org.

[B10] Potsdam. http://www.uni-potsdam.de/over/homegd.htm.

[B11] INIST. http://www.inist.fr/index_en.php.

[B12] e-Depot. http://www.kb.nl/.

[B13] Lawrence S (2001). Free online availability substantially increases a paper's impact. Nature.

[B14] Velterop J (2003). Should scholarly societies embrace Open Access (or is it the kiss of death)?. Learned Publishing.

[B15] Open Access law introduced. http://www.the-scientist.com/news/20030627/04.

[B16] Tan-Torres Edejer T (2000). Disseminating health information in developing countries: the role of the internet. BMJ.

